# ROS-Centered Transcriptomic Regulatory Networks Linking Salinity Stress, Antioxidant Defense and Processability Traits in *Salicornia* spp.

**DOI:** 10.3390/cimb48070719

**Published:** 2026-07-15

**Authors:** Nurtai Gubaidullin, Gulnazym Ospankulova, Aisarat Gajimuradova, Alfiya Syzdykova, Aibek Zhumalin, Kalamkas Dairova, Damilya Konysbayeva, Viktoriya Gorbulya, Kadyrzhan Makangali

**Affiliations:** 1 Institute of Animal Science and Veterinary Medicine, Saken Seifullin Kazakh Agrotechnical University, Astana 010000, Kazakhstan; nur-tai.kz@mail.ru (N.G.); halik.kz@mail.ru (A.S.); aibek_biotex@mail.ru (A.Z.); 2 Institute of Engineering and Food Technology, Saken Seifullin Kazakh Agrotechnical University, Astana 010000, Kazakhstan; kalamkasdairova685@gmail.com; 3 Engineering Center for Organic Agricultural Technology, Saken Seifullin Kazakh Agrotechnical University, Astana 010000, Kazakhstan; aisarat3878@mail.ru; 4 Institute of Agriculture and Forestry, Saken Seifullin Kazakh Agrotechnical University, Astana 010000, Kazakhstan; damilya_konysbaeva@mail.ru (D.K.); v.gorbulya@kazatu.edu.kz (V.G.)

**Keywords:** salinity stress, halophytes, *Salicornia* spp., transcriptomics, RNA-seq, regulatory networks, gene expression, abiotic stress, omics approaches, processability characteristics

## Abstract

Salinity stress affects not only the survival and productivity of halophytic plants, but also the composition, structure and processability of their biomass. In *Salicornia* spp., salt-induced regulation of ion transport, osmotic adjustment, reactive oxygen species signaling, antioxidant defense, and cell wall remodeling can directly influence residual salinity, water retention, texture, extractability, drying behavior, and oxidative stability of plant biomass. However, most existing transcriptomic studies of *Salicornia* and related halophytes have focused mainly on salt tolerance mechanisms, whereas the connection between stress-regulated molecular networks and processing-related biomass traits remains insufficiently systematized. This review addresses this gap by proposing a mechanistic framework that links salinity perception, ROS-mediated signaling, ABA and MAPK pathways, antioxidant gene families, transcription factor networks and processing-oriented quality traits. Special attention is given to enzymatic antioxidant systems, including SOD, CAT, APX, POD and components of the ascorbate-glutathione cycle, as well as to non-enzymatic defense mechanisms involving ascorbate, glutathione, phenolic compounds, carotenoids, proline and glycine betaine. The review also discusses the regulatory roles of WRKY, DREB/CBF, NAC, bZIP and MYB transcription factor families as molecular control points connecting salinity stress responses with downstream metabolic and structural traits. Network-based approaches, including WGCNA, pathway signatures and transcript panels, are considered more informative than single-gene markers for predicting complex quality traits in *Salicornia* biomass. In addition, recent genomic and computational strategies, including CRISPR/Cas-mediated functional validation, GWAS, genomic selection, multi-omics integration and AI-assisted modeling, are discussed as emerging tools for candidate-gene prioritization and predictive assessment of stress-dependent biomass quality. Overall, this review shifts the interpretation of *Salicornia* transcriptomics from a descriptive salt-tolerance model toward a mechanistic and application-oriented framework for improving halophytic raw materials for food, feed and bioprocessing applications.

## 1. Introduction

Global climate change and the increasing frequency of extreme weather events intensify inter-seasonal and inter-regional variability in the quality of plant raw materials. Fluctuations in temperature, water availability, mineral nutrition, cultivation conditions, and storage history affect fraction yield, rheological behavior, starch pasting properties, protein and lipid extractability, and resistance to post-harvest defects [1,2,3,4]. For industrial processing, this creates practical uncertainty, because batches with similar external characteristics may behave differently during milling, hydration, heating, enzymatic treatment or extraction. Additional variability is introduced by processing itself, since the functional properties of plant ingredients depend not only on the original raw material, but also on the method of isolation and the structural state of the component after pretreatment [5].

Processing quality is usually assessed by instrumental and laboratory tests selected according to the crop and processing purpose [6]. These tests are necessary, but they mainly describe the final phenotype and provide limited information about the molecular causes of variation between genotypes, seasons and batches. As a result, quality management often remains reactive: deviations are detected during acceptance or after processing, when opportunities for correction are already limited [7,8]. A transition to earlier prediction requires molecular indicators that connect genotype, environment, development, and storage conditions with functional tissue properties. In this context, gene expression can be considered an informative intermediate phenotype that reflects both the genetic program and the physiological state of the raw material [9,10].

The mechanistic link between transcriptomic variation and technological behavior is based on the coordinated regulation of pathways involved in the synthesis and remodeling of starch, storage proteins, cell wall polysaccharides, lipids, and secondary metabolites. Changes in the expression of genes encoding enzymes and regulatory proteins can alter biopolymer composition, tissue structure and processing behavior. For starchy crops, this may affect amylose and amylopectin balance, chain architecture, granule crystallinity and gelatinization profile [11,12,13]. For cereals, expression programs related to endosperm texture and protein matrix organization influence hardness, starch damage during milling and water absorption [4]. However, transcriptomic interpretation has important limitations. mRNA abundance does not always correspond to protein amount or activity because of translation efficiency, protein stability, post-translational regulation and intracellular localization [14,15]. In addition, bulk RNA-seq averages signals across mixed cell types, which may hide tissue-specific regulatory events, especially in structurally heterogeneous organs [16].

Therefore, the most useful approach for applied quality research is the integration of transcriptomic data with quantitative technological phenotyping and regulatory network analysis. This makes it possible to move from isolated gene associations to more stable markers, such as co-expression modules, pathway signatures and compact transcript panels [17]. Such markers are especially relevant for *Salicornia perennis*, where processing quality is strongly shaped by salinity-dependent regulation of ion homeostasis, osmoprotection, antioxidant defense, lipid metabolism and cell wall remodeling. Despite the increasing number of transcriptomic studies on halophyte salt tolerance, several important gaps remain unresolved. First, most available studies describe differentially expressed genes under salinity stress but do not integrate them into mechanistic regulatory networks that connect ROS signaling, antioxidant defense, transcription factor activity and downstream metabolic remodeling. Second, gene-family-level interpretation remains limited, especially for antioxidant enzymes such as SOD, CAT, APX, and POD, and for transcription factor families such as WRKY, DREB/CBF, NAC, bZIP, and MYB. Third, the technological relevance of these molecular responses is still poorly systematized, although salinity-regulated processes can influence residual salt content, water retention, tissue firmness, drying stability, extractability and oxidative stability of *Salicornia* biomass. Finally, emerging approaches such as CRISPR/Cas-based functional validation, GWAS, genomic selection, multi-omics integration and AI-assisted modeling have not yet been sufficiently incorporated into a unified framework for predicting stress-dependent biomass quality.

Therefore, the aim of this review is to synthesize current knowledge of salinity-responsive transcriptomic regulatory networks in *Salicornia* spp. and to propose a mechanistic framework linking ROS-centered stress signaling, antioxidant defense, transcription factor regulation, and processing-oriented processability traits of halophytic biomass.

## 2. Processability Characteristics of Plant Raw Materials as Outputs of Transcriptome Programs

Industrial processing of plant raw materials depends on specific technological indicators rather than on a general concept of quality. These indicators include fraction yield, extractability of proteins, oils, and polysaccharides, hydration and heating behavior, oxidative and enzymatic stability, and rheological properties under mixing or shear. Differences in these traits are partly determined by the molecular organization of raw material components, including protein structure, starch type, lipid composition and dietary fiber properties [5,18,19].

**Figure 1:**
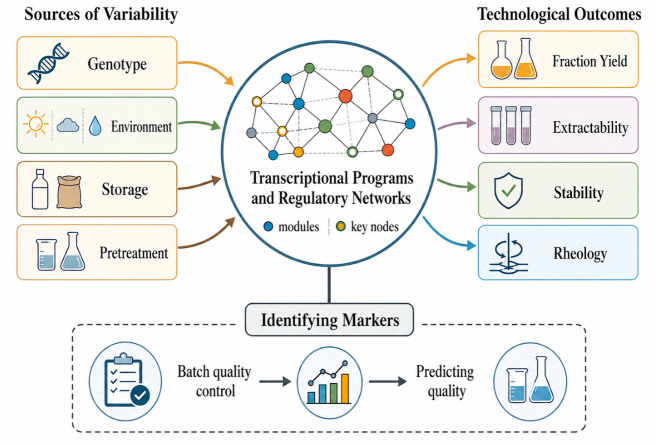
Conceptual framework connecting transcriptional regulatory networks with processability characteristics of plant raw materials.

The attempt to explain processability characteristics through molecular mechanisms faces two key methodological difficulties (Figure 1).

Two methodological difficulties limit the direct interpretation of transcriptomic markers. The first is multilevel causality. Gene expression is an intermediate layer between genotype, environment, and tissue properties. It is influenced by genetic background, growth conditions, developmental stage, storage and pre-processing history [20,21]. Therefore, transcriptomic data can support prediction and mechanism discovery, but they do not prove causality by themselves. Key associations should be interpreted together with regulatory networks and validated at the protein, metabolite or processing phenotype level [14].

The second difficulty is scale mismatch. Processability characteristics are determined by tissue microstructure and the chemical organization of biopolymers, including starch granule damage, beta-glucan molecular weight, pectin methylation, and the spatial distribution of proteins and lipid bodies [22,23]. Bulk transcriptomic data may hide tissue-specific events, especially in heterogeneous organs. Therefore, transcriptomic markers should be used as mechanistic indicators, not as substitutes for chemical and processing measurements [24,25].

Thus, transcriptome markers of processability characteristics should be considered not as a substitute for chemical and processing measurements, but as a tool for explaining mechanisms. They help build hierarchical models that link the genetic program, environmental factors, and biochemical organization of tissues to the final parameters of industrial processing of plant raw materials [26,27].

### 2.1. Conceptual Model: ‘Gene Expression → Pathways → Composition and Structure of Biopolymers → Processing Parameters’

The proposed model links transcriptomic variation with processability characteristics through several intermediate levels. In simplified form, the chain can be described as gene expression → pathway activity → composition and structure of biopolymers → measurable processing parameters. However, this chain should not be interpreted as strictly linear. Growing conditions, storage regime and technological pre-processing can modify each transition, which is why direct associations between a transcript and a technological trait are often unstable.

The first critical transition is the shift from mRNA abundance to the activity of enzymes and regulatory proteins. At this level, translation efficiency, protein stability, post-translational modification, and intracellular localization may weaken the relationship between transcript level and functional activity [28,29]. The second transition connects enzyme activity with polymer structure. Here, substrate availability, compartmentalization, transport processes and the timing of pathway activation are important [30,31]. The third transition links polymer structure with the final technological parameter. At this stage, processing conditions become especially influential. Temperature, moisture, pH, mechanical load and storage duration can change how the same biochemical difference appears in viscosity, texture, extractability, browning or stability [32,33,34].

A clear example is cold-induced sweetening in potato tubers. Suppression of the vacuolar invertase transcript VInv reduces the accumulation of reducing sugars during cold storage and leads to lighter fried products with lower acrylamide formation [35]. This case shows an almost complete chain from gene expression to metabolic composition and subsequently to processing quality. At the same time, later studies indicate that the strength of this effect depends on storage conditions and the initial metabolic state of the tuber [36]. Therefore, even a strong candidate gene requires standardized sampling and storage protocols before it can be used as a reliable quality marker.

A second example is wheat grain hardness. Variation in puroindoline genes is associated with endosperm texture, which affects grain breakage during milling, starch damage, and flour water absorption [4]. Experimental overexpression of Pina in durum wheat reduced grain hardness, decreased starch damage, and changed flour water binding [37]. This case demonstrates that a transcriptomic or genetic signal becomes technologically meaningful only when it is connected to a defined phenotyping protocol. Milling conditions, grain moisture, and conditioning temperature can modify the final effect, so molecular markers must be interpreted together with standardized technological testing.

For more complex traits, single genes are usually insufficient. In rice quality, malting performance of barley, and gluten-related wheat rheology, technological behavior is shaped by coordinated changes in starch metabolism, cell wall remodeling, reserve protein accumulation, and regulatory networks [38,39,40,41,42,43,44,45,46]. These examples support the use of pathway signatures, co-expression modules and small transcript panels rather than isolated genes. Such markers better reflect the polygenic and context-dependent nature of processing traits.

This logic is especially important for halophytic raw materials such as *Salicornia*. In these plants, transcriptomic programs are strongly affected by salinity, ion homeostasis, osmoprotection, antioxidant defense, and carbon allocation [47,48]. These processes can change tissue hydration, cell wall properties, lipid composition, extractability and drying stability. In addition, the rhizosphere microbiota may further modify stress responses and secondary metabolism [49]. Therefore, for *Salicornia*, transcriptomic markers should not be transferred between experiments unless salinity level, developmental stage, tissue type and sampling conditions are strictly described. The most appropriate marker format is not a single transcript, but a modular signature that captures the coordinated response of salt adaptation pathways linked to processing quality.

### 2.2. The Role of Regulatory Networks and the Significance of RNA-Seq

In studies of processability characteristics of plant raw materials, regulatory networks describe how transcription factors, target genes, small RNAs and co-expressed gene groups coordinate the biological processes that shape processing quality [50,51,52,53]. This approach is more informative than the analysis of individual differentially expressed genes, because many processability traits depend on the coordinated regulation of starch metabolism, storage protein accumulation, lipid biosynthesis, cell wall remodeling and stress responses [54,55]. Network-based analysis, including WGCNA, TF-target relationships and regulatory motif assessment, helps identify modules and hub genes that may serve as markers or control points for quality-related traits [56,57,58,59]. The choice of RNA-seq strategy determines the level of interpretation. Bulk RNA-seq is most suitable for comparing genotypes, growing conditions and raw material batches, because it provides an integrated expression profile that can be used for marker discovery and batch quality prediction [60,61]. However, bulk data average signals across mixed cell types and may miss local regulatory events. Single-cell and spatial transcriptomics are therefore more useful for mechanistic studies, where it is necessary to determine which cell types or tissue zones form a quality-related signal [16,62]. Small RNA-seq adds another regulatory layer by revealing miRNA and siRNA pathways that can modify mRNA abundance and affect the accumulation of structural and metabolic components [63]. Thus, bulk RNA-seq is practical for transferable quality markers, whereas single-cell, spatial and small RNA approaches are mainly needed to explain the mechanisms behind those markers [10,64,65].

## 3. Study Design and RNA-Seq Data Acquisition for Quality and Raw Material Processing

The reliability of transcriptomic markers for raw material quality depends primarily on the design of the RNA-seq study. A marker does not describe quality in general, but reflects the physiological state of a defined tissue at a defined developmental or storage stage. Therefore, tissue selection must be guided by the technological trait under investigation. For grain fractionation and rheology, the most informative tissues are those involved in endosperm structure, protein matrix formation and starch deposition, because these determine milling behavior, starch damage, water absorption and flour functionality [66,67]. For oilseed crops, sampling should focus on tissues where storage lipids and proteins accumulate, whereas in fruits and vegetables the most relevant tissues are those controlling texture, water movement, cell wall remodeling and post-harvest defects [68,69,70]. In traits linked to reserve accumulation, sampling during active synthesis and matrix restructuring is usually more informative than sampling at a late static stage. For example, in soybean, the period of intensive seed weight increase and oil accumulation is associated with activation of carbon metabolism and lipid biosynthesis pathways, which makes it suitable for connecting expression patterns with processing-related phenotypes [71].

A second requirement is the control of biological variation. Transcriptomic markers intended for quality prediction must remain informative across genotypes, seasons, growing conditions, and storage regimes. This is especially important because genotype and environment interactions can strongly modify expression profiles and may mask stable regulatory signals. Multi-season designs and the integration of RNA-seq with genetic approaches such as GWAS and TWAS help separate expression changes related to the genetic basis of a trait from temporary responses to local conditions [72,73]. For fruits, vegetables and storage organs, post-harvest regimes must be treated as major design factors, since cold storage, dehydration, hormonal treatments and mechanical damage can induce independent transcriptional programs that affect texture, browning, water retention and metabolic stability [74].

Technical variation must also be minimized from the beginning of the experiment. Batch effects can arise during sampling, RNA extraction, library preparation, and sequencing. They become especially problematic when technical batches coincide with biological groups, because the resulting signal may appear to be a quality marker while actually reflecting laboratory handling [75]. For this reason, samples should be randomized across extraction, library preparation and sequencing batches. Biological replication is also essential. Empirical work on RNA-seq indicates that three biological replicates are often insufficient for robust detection of expression differences, especially when effects are moderate or small [76]. When resources are limited, increasing biological replication is usually more useful than only increasing read depth after an adequate sequencing level has already been reached [77].

RNA extraction is more of a design element than a purely technical step. Plant raw materials rich in oils, polysaccharides, phenolic compounds or dense seed matrices may inhibit extraction and reduce library quality. Protocols based on CTAB, PVPP, LiCl precipitation and additional purification steps are often used for difficult tissues [78,79,80]. However, the most important requirement for marker development is not the protocol name itself, but complete documentation of RNA integrity, storage before extraction and all handling conditions. For review-level recommendations, these details can be summarized as minimum design requirements rather than described as a laboratory protocol.

## 4. Analytical Strategies: From Read-Level Analysis to Functional Pathways and Regulatory Insights

The analytical strategy in RNA-seq studies of raw material quality should be aimed not only at detecting differentially expressed genes, but at identifying expression signals that can be mechanistically connected with processability traits. In this context, quality control is the first biological filter. Systematic differences in read quality, duplication, adapter contamination, mapping rate, or library composition may be incorrectly interpreted as genotype, salinity, or processing effects. Therefore, quality metrics should be assessed across all samples simultaneously, for example using consolidated reports such as MultiQC, because this makes it possible to detect batch-related shifts before marker discovery [81]. After quality control, reads are transformed into gene or transcript expression matrices. For applied prediction of raw material behavior, gene-level quantification is usually more stable, while transcript-level or isoform-level analysis is more useful when the hypothesis concerns alternative splicing, enzyme localization or structural protein variants [82,83,84,85,86].

Normalization and statistical modeling determine whether weak but coordinated biological signals can be detected reliably. This is especially important for processability traits, because they are rarely explained by one strongly changing transcript. More often, processing behavior reflects moderate coordinated changes in pathways controlling starch metabolism, cell wall remodeling, lipid biosynthesis, protein accumulation, ion transport or stress protection. Therefore, expression values used for descriptive profiling should not be mixed with count-based statistical models used for differential analysis. Appropriate models should account for library size, since differences in sequencing depth can artificially alter read counts and generate false differences in expression [87,88]; compositional effects, where the overrepresentation of specific transcripts distorts the relative distribution of reads within a library and reduces the comparability of simple normalized measures such as RPKM/FPKM [87,89]; and biological variability between replicates, which is required for reliable dispersion estimation and for distinguishing true effects of genotype, tissue, or salinity stress from random fluctuations in expression [88,90]. Differential expression analysis should be treated as the first stage of candidate discovery, not as proof of technological relevance. A gene becomes meaningful only when its expression change can be linked to a pathway, a tissue process, and a measured processing trait.

Functional interpretation is the step where transcriptomic data begin to acquire technological meaning. GO, KEGG, and MapMan help assign genes to biological processes and plant metabolic categories, but the most useful interpretation for quality studies is pathway-level analysis rather than isolated gene annotation [91,92,93]. Gene set and enrichment approaches are valuable because they can detect coordinated shifts even when individual genes show moderate changes [94,95]. For *Salicornia*, this is particularly important. A group of transcripts related to ion transport and vacuolar compartmentalization may indicate changes in residual salinity and tissue water status. Osmoprotection and antioxidant modules may be linked with dehydration resistance, color preservation and storage stability. Cell wall remodeling modules may explain tissue firmness, grindability and extractability. Lipid metabolism modules may be associated with membrane stability and oxidative resistance of biomass. Thus, the analytical goal is to move from a gene list to pathway signatures that can be compared with real processing measurements.

Annotation quality remains a major limitation. In plants, gene duplication, paralogues, and incomplete genome annotation can distort pathway assignment and reduce marker transferability [96,97,98,99,100]. This problem is especially relevant for *Salicornia*, where genomic resources are less developed than in major crops. Therefore, conclusions are usually more reliable at the level of conserved pathways and co-expression modules than at the level of individual transcripts or exact transcription factor target interactions [101,102]. For *Salicornia*, this means that markers should preferably be selected as module signatures of salinity-regulated processes, rather than as single candidate genes transferred directly from other species.

Reproducibility should be considered part of marker validation. RNA-seq studies must report the reference genome and annotation version, mapping or quantification strategy, normalization method, statistical model, software versions, and criteria for sample inclusion. Public deposition of raw reads and metadata in repositories such as GEO or SRA allows later reanalysis and comparison across genotypes, seasons, salinity regimes and laboratories [103,104]. For processing-oriented studies, transcriptomic signatures should also be compared with proteomic, metabolomic and technological data when possible [105]. Only under these conditions can an expression signature be evaluated not merely as a statistical result, but as a potential marker for predicting raw material quality under independent processing conditions.

**Figure 2:**
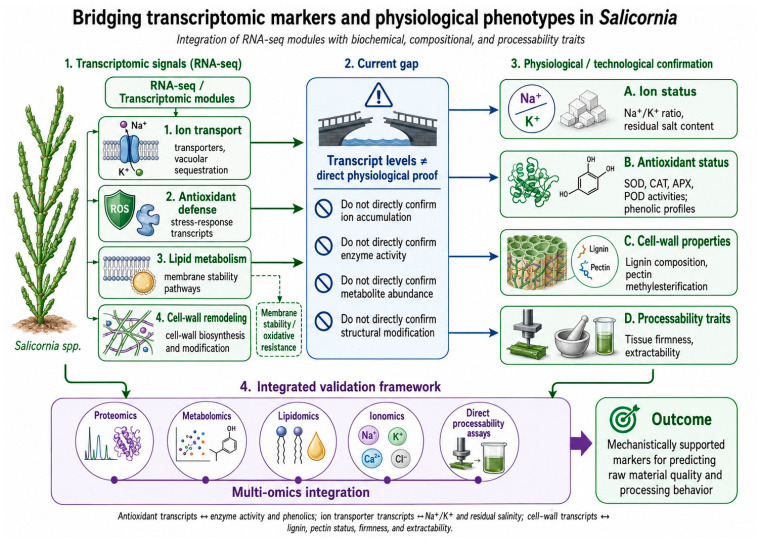
Integration of transcriptomic markers and physiological phenotypes in *Salicornia*.

A significant gap persists between transcriptomic markers and physiological phenotypes in *Salicornia* research (Figure 2).

While transcriptomic data can signal the activation of ion transport, antioxidant defense, lipid metabolism, or cell-wall remodeling, they fail to confirm actual ion accumulation, enzyme activity, metabolite abundance, or structural modifications. Consequently, transcriptomic modules must be integrated with proteomics, metabolomics, lipidomics, ionomics, and direct processability assays. For instance, antioxidant transcripts should be correlated with SOD, CAT, APX, or POD activity and phenolic profiles; ion transporter transcripts with Na^+^/K^+^ ratios and residual salt content; and cell-wall-related transcripts with lignin composition, pectin methylesterification, tissue firmness, and extractability.

## 5. Transcriptomic Markers of Processing Traits: Identification, Prioritization and Validation

Transcriptomic markers of processing traits should be interpreted as biological indicators that connect gene expression with a measurable processing phenotype. Their value depends not only on statistical significance, but also on mechanistic plausibility, stability across conditions, and the possibility of conversion into a practical assay. In this context, marker discovery should not end with a list of differentially expressed genes. A candidate becomes useful only when it can be placed into a pathway, linked with a technological trait and validated on independent material.

### 5.1. Marker Formats and Their Biological Meanings

The simplest format is a single gene marker. Such markers are most convincing when the technological trait is controlled by a relatively direct biochemical step. A good example is cold-induced sweetening in potato, where vacuolar invertase activity is associated with reducing sugar accumulation during storage. Tai et al. showed that potato cultivars with different cold induced sweetening responses have distinct tuber transcriptomic profiles, including genes related to carbohydrate metabolism [106]. Together with earlier functional evidence on vacuolar invertase, this supports a clear mechanistic chain: expression of sugar metabolism genes, accumulation of reducing sugars, dark frying color and higher acrylamide risk. This type of marker is strong because the pathway is biologically interpretable. However, it is also narrow. It works best for specific storage defects or traits dominated by one biochemical control point, but it is less suitable for complex traits such as texture, rheology, extractability or biomass stability. For polygenic traits, transcript panels are usually more reliable than individual genes. A panel combines several transcripts from the same pathway or from a trait associated co-expression module. This format is biologically justified when the processing phenotype depends on coordinated regulation of several enzymes and structural proteins. For example, Hadish et al. developed transcriptomic biomarkers for apple firmness and postharvest fruit quality, showing that prediction of texture is more realistic when multiple transcripts are considered rather than a single candidate [107]. This observation indicates that firmness is not formed by one gene; it reflects cell wall metabolism, pectin modification, water status and ripening related regulation. Therefore, a transcript panel is more suitable for traits where several pathways converge on one technological property.

A more integrative format is the expression signature score. In this approach, a predefined gene set is summarized into one quantitative index representing pathway activity. This logic is close to gene set enrichment analysis and single sample scoring approaches, where the pathway itself becomes the phenotype [94,108]. For raw material control, this is useful because industrial decisions are often made for one incoming batch, not for a group comparison. A signature score can therefore indicate whether a batch has a high or low activity of a biologically meaningful process, such as antioxidant defense, cell wall remodeling, lipid biosynthesis, or osmotic adaptation. The weakness of this approach is that the result depends on the selected gene set and annotation quality. If the pathway definition is incomplete or transferred from a poorly related species, the score may become statistically stable but biologically misleading.

Predictive models represent the most applied format. Azodi et al. showed that transcriptome-based models can predict complex traits in maize at a level comparable with genetic marker-based models [109]. This work is important because it demonstrates that expression data can contain strong predictive information. However, the same study also illustrates the central problem of machine learning markers: high prediction accuracy does not automatically mean mechanistic understanding. A model can predict well inside one dataset but fail when genotype, season, tissue stage or laboratory protocol changes. For processing quality studies, predictive models should therefore be used together with interpretable modules and pathway information, not as black box substitutes for biological reasoning.

**Figure 3:**
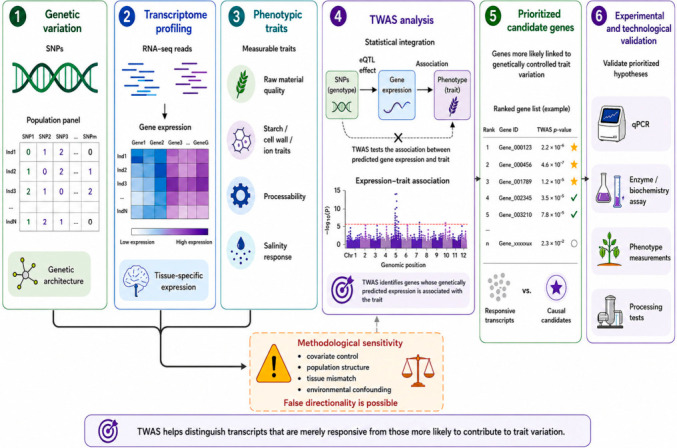
Conceptual framework of the TWAS approach for candidate gene prioritization.

TWAS provides another strategy for prioritizing candidate genes (Figure 3).

TWAS enables the prioritization of candidate genes by integrating expression variation with the genetic architecture of a trait, thereby strengthening gene-to-phenotype discovery and helping to move from broad association signals to biologically interpretable genes [110]. The practical value of this approach has been demonstrated in rice, where the combination of GWAS and transcriptomic analysis identified candidate genes associated with starch pasting properties, an important technological trait related to grain quality [111]. However, TWAS does not provide direct evidence of causality; its results may be influenced by covariate selection, population structure, tissue mismatch, and environmental confounding, which can lead to false associations or incorrect directionality [110]. Therefore, TWAS-derived candidates should be considered prioritized hypotheses that require further experimental and technological validation [110,111].

### 5.2. Prioritization Criteria for Processing Markers

For processing-oriented marker discovery, candidate prioritization should follow four criteria. The first criterion is effect size. A marker should show a measurable relationship with the technological trait, not only a statistically significant difference. In large datasets, very small expression changes may become significant but remain useless for quality control. The second criterion is biological coherence. The candidate should belong to a pathway that can reasonably explain the phenotype. For example, cell wall remodeling genes are plausible candidates for firmness, grindability and extractability, whereas ion transport and osmoprotection genes are more relevant for water retention and salinity dependent biomass quality.

The third criterion is stability. A useful marker must retain its direction and predictive value across genotypes, seasons, locations, storage conditions, and technical batches. This is the main difference between an experimental association and a practical quality marker. Qiao et al. discussed transcriptomics as a tool for crop improvement, but their analysis also supports the view that expression markers must be tested across biological contexts before they can be considered transferable [60]. The fourth criterion is technical robustness. Batch effects can create false markers when RNA extraction, library preparation or sequencing batches coincide with biological groups [112,113,114]. Therefore, marker selection must include randomization, batch diagnostics and independent validation.

### 5.3. Validation of Transcriptomic Markers

Validation should be considered a separate stage of marker development, not a final formality. The minimum standard is an independent test set that differs from the discovery set by season, genotype, location, storage regime, or processing batch. The stronger option is validation in another laboratory or with independently prepared RNA libraries, because this tests whether the marker survives technical variation. Public repositories such as GEO and SRA make this possible when raw reads and metadata are available [104]. For practical use, transcriptomic markers should be converted into targeted assays. qPCR panels are the most realistic format for routine testing because they allow several transcripts to be measured with lower cost and simpler interpretation than whole transcriptome sequencing. However, qPCR validation requires stable reference genes, RNA quality control and calibration against real processing measurements [115,116]. A panel that predicts expression differences but is not calibrated against firmness, extract yield, viscosity, drying loss or residual salinity has limited industrial value. Therefore, the correct validation chain should be RNA-seq discovery, candidate prioritization, targeted assay development, independent biological validation and direct comparison with technological phenotyping.

### 5.4. Application to Raw Material Quality Prediction

The main application of transcriptomic markers is early prediction of raw material behavior before full processing. Hadish et al. used transcriptomic biomarkers to predict apple firmness, which is conceptually close to incoming quality control because the aim is to estimate future postharvest performance before quality loss becomes visible [107]. Azodi et al. showed that transcriptome-based prediction can model complex traits, but their results also indicate that prediction should be tested beyond the training population [109]. Torres-Rodriguez et al. and Lee et al. further show that integrating expression with genetic information can improve candidate prioritization for complex quality traits, especially when the phenotype depends on coordinated metabolic regulation [110,111].

A scientifically justified strategy is a hierarchy of markers. Single gene markers should be used only when the causal pathway is narrow and experimentally supported. Transcript panels are preferable for processability traits that are controlled by several related genes. Signature scores are useful when the pathway is more important than any single transcript. Predictive models can be applied when large, well-annotated training datasets are available, but they require strict external validation. TWAS candidates are valuable for mechanistic prioritization, but they should not be treated as final functional proof. This hierarchy is especially important for *Salicornia*, where processing quality is likely to depend on coordinated modules of ion transport, osmoprotection, antioxidant defense, lipid metabolism and cell wall remodeling rather than on one dominant transcript.

## 6. Regulatory Expression Networks: Modules, Key Nodes and Molecular Control Points

Regulatory network analysis is needed because most processability traits of plant raw materials are not controlled by one transcript, but by the coordinated regulation of metabolic and structural pathways. Differential expression analysis can show which genes change between samples, but it does not explain whether these genes act together or whether they belong to the same biological process. In contrast, WGCNA groups genes into co-expression modules according to similarity of expression patterns and then summarizes each module by a module eigengene, which reflects the overall activity of that module [117]. This is important for quality research because the module eigengene can be directly correlated with quantitative traits such as oil content, cell wall stiffness, extractability, texture, viscosity, stress tolerance or drying stability. In this sense, WGCNA converts thousands of individual transcripts into biologically interpretable units that can be compared with processing parameters.

The strength of WGCNA is well illustrated by Yao et al., who used this approach in soybean and identified modules associated with seed size and oil content during specific developmental stages [71]. Their work is important not only because it identified hub genes, but also because it showed that the relationship between expression and quality traits is stage dependent. In other words, a marker may be informative only during the period when the corresponding biochemical pathway is actively forming the future property of the raw material. This is directly relevant to processing studies: for lipid-rich materials, the best markers are expected during active lipid biosynthesis, while for biomass texture or extractability, the most informative window may coincide with cell wall formation and remodeling. Similar logic is discussed by Panahi et al. for barley malting quality, where extract yield, wort viscosity and fermentation performance are not explained by one gene, but by coordinated modules related to reserve mobilization, cell wall degradation and hormonal regulation [38]. Therefore, WGCNA should be used in processing quality studies as a tool for identifying pathway-level predictors that are closer to processing behavior than isolated differentially expressed genes.

However, co-expression modules alone do not prove regulation. Genes may be co-expressed because they are controlled by the same upstream factor, because they respond to the same environmental signal, or because they are indirectly connected through developmental stage or batch effects. This limitation is critical for applied quality studies. For example, a module correlated with texture or extractability may include true regulators of cell wall remodeling, but it may also include secondary stress-response genes that only reflect the physiological state of the tissue. Therefore, module interpretation must combine three levels of evidence: correlation with the technological trait, functional enrichment of the module, and biological plausibility of candidate regulatory genes.

TF-target analysis provides the next level of interpretation. It helps test whether transcription factors inside or outside a module may regulate structural or metabolic genes responsible for the trait. PlantRegMap web platform provides predicted plant regulatory interactions and transcription factor binding information, while DAP-seq data from O’Malley et al. provide experimental evidence of transcription factor binding sites in Arabidopsis that can be cautiously used through orthology and motif conservation [55,118]. Compared with WGCNA, TF-target analysis is more directional because it tries to distinguish potential regulators from downstream genes. However, this directionality remains a hypothesis unless it is supported by motif evidence, binding data, perturbation experiments or independent validation. Network reconstruction algorithms such as GENIE3, ARACNe and CLR can help infer regulatory relationships from expression data, but Huynh-Thu et al. clearly showed that such methods reconstruct statistical dependencies, not direct causal mechanisms [119]. Therefore, they should be used for prioritization, not as final proof.

Hub genes are the most practical output of network analysis. In WGCNA, hub genes are usually selected by high intramodular connectivity and strong correlation with the module eigengene [117]. In processing quality research, a strong candidate hub should satisfy several conditions. First, it should belong to a module significantly associated with a measured processing trait. Second, the module should be enriched for a biologically relevant pathway, such as lipid biosynthesis, cell wall modification, ion transport, osmoprotection or antioxidant defense. Third, the hub should have functional annotation or regulatory evidence that explains its possible role in the trait. Fourth, the relationship should be reproducible in independent samples, seasons or experimental conditions. Without these criteria, a hub gene may simply be a mathematically central transcript rather than a functional control point.

For *Salicornia*, this network logic is particularly important because salinity can activate many genes that are not directly responsible for processing quality. A gene that responds strongly to salt is not automatically a useful processing marker. The more valuable candidates are hubs within modules that connect salt response with measurable biomass properties, such as water retention, residual salinity, tissue firmness, drying stability, oxidative stability and extractability. For example, modules enriched in ion transport and vacuolar compartmentalization may be linked with tissue hydration and residual salt accumulation; modules enriched in osmoprotective and antioxidant pathways may be linked with dehydration tolerance and storage stability; modules enriched in lignin biosynthesis, pectin modification and cell wall remodeling may be linked with tissue firmness, grindability and extraction efficiency. Thus, the best *Salicornia* markers should be selected not only by differential expression under salt stress, but also by their position in trait-associated modules and by their possible regulatory connection to processing-relevant pathways.

Overall, regulatory network analysis provides a rational filter between transcriptome data and practical marker development. WGCNA identifies modules associated with processability traits, TF-target analysis suggests possible regulatory directions, and hub-gene prioritization reduces the number of candidates for validation. At the same time, network analysis does not replace experimental proof. A regulatory candidate becomes scientifically convincing only when its expression is reproducible, its module is linked with a real processing phenotype, its pathway role is biologically plausible, and its effect is supported by independent transcriptomic, proteomic, metabolomic or functional data. This framework is appropriate for moving from large RNA-seq datasets to compact marker panels for processing-oriented quality assessment of plant raw materials.

## 7. ROS-Centered Signaling Under Salinity and Related Stress Responses

ROS are common signaling and stress-response molecules in plants under salinity, drought, temperature stress, and biotic interactions. Their role depends on concentration, duration and site of formation: excessive ROS cause lipid peroxidation, protein oxidation, pigment degradation and membrane damage, whereas controlled ROS production participates in Ca^2+^ signaling, ABA-dependent responses, MAPK cascades and transcriptional reprogramming [120,121,122]. Therefore, ROS-related transcripts should not be interpreted only as markers of oxidative injury, but also as components of shared stress-signaling networks.

Salinity and drought are closely connected because both disturb cellular water balance and induce ROS formation through changes in photosynthesis, respiration and membrane homeostasis. However, salinity additionally causes ionic stress through Na^+^ and Cl^−^ accumulation, which activates ion transport, vacuolar sequestration and Na^+^/K^+^ homeostasis pathways [1,123]. This distinction is important for *Salicornia*, where Na^+^ accumulation and vacuolar compartmentalization are part of the adaptive strategy. Heat and cold stress can also increase ROS formation through disturbed electron transport and membrane instability, whereas pathogen recognition often induces an apoplastic ROS burst as part of defense signaling [120,124]. Thus, ROS connect different stress categories, but their upstream source and biological meaning depend on the type of stress.

A common regulatory mechanism is the Ca^2+^–ROS feedback loop. Stress-induced Ca^2+^ changes activate Ca^2+^-dependent kinases and RBOH proteins, leading to controlled ROS production and MAPK activation [125]. For transcriptomic analysis, genes encoding Ca^2+^ sensors, CDPKs, RBOH proteins, MAPKs, and antioxidant enzymes should be analyzed as connected modules rather than as separate stress categories. In *Salicornia*, this is particularly important because ROS signaling interacts with ion transport, osmoprotection, antioxidant defense and tissue succulence.

At the gene-family level, antioxidant defense is mainly controlled by SOD, CAT, APX, POD/GPX and enzymes of the ascorbate-glutathione cycle, including GR, MDHAR and DHAR. These families have different biochemical roles. SOD converts superoxide radicals into H_2_O_2_, whereas CAT, APX and POD remove H_2_O_2_ through different cellular routes. CAT is mainly associated with rapid H_2_O_2_ removal, APX with ascorbate-dependent redox protection in chloroplasts, cytosol, and mitochondria, and POD with both H_2_O_2_ detoxification and cell wall oxidation, lignification, and cross-linking [126,127,128]. Therefore, the induction of one antioxidant gene family does not necessarily indicate complete oxidative protection. A more informative marker is the balance between SOD-generated H_2_O_2_ and its removal through CAT-, APX- and POD-dependent pathways.

This distinction is important for *Salicornia* because comparative studies show that antioxidant enzymes do not contribute equally to salt tolerance. In *S. persica*, *S. europaea,* and *S. bigelovii*, APX and POD activities were important components of the antioxidant response under moderate and high salinity, while *S. persica* showed higher tolerance associated with stronger antioxidant and osmoprotective responses [129]. Earlier work on *S. persica* and *S. europaea* also showed species-dependent changes in SOD, CAT, APX and peroxidase activities under NaCl treatment [130]. Thus, antioxidant gene families should be interpreted not only as general ROS-scavenging markers, but also as species-dependent components of salinity adaptation.

ABA provides the hormonal connection between osmotic stress and transcriptional regulation. Under salinity, ABA signaling activates SnRK2 kinases and ABF/AREB-type bZIP transcription factors, while ABA-induced ROS production can reinforce Ca^2+^ signaling and downstream kinase activity [131,132]. In this system, ROS and ABA form a partially overlapping feedback network: ABA promotes adaptive stress signaling, ROS transmit and amplify the signal, and antioxidant enzymes prevent uncontrolled oxidative damage. For RNA-seq studies, upregulation of SOD, CAT, or APX may therefore reflect not only oxidative injury, but also active ABA-dependent stress regulation.

MAPK cascades add another regulatory layer between ROS perception and gene expression. In Arabidopsis and other model plants, MAPK modules such as MKK2–MPK4/MPK6 and MPK6-related pathways have been linked with salt, osmotic and oxidative stress responses [133,134]. MAPKs can regulate transcription factors, antioxidant genes, hormone signaling, and cell wall-related processes. Therefore, MAPK-related transcripts are useful mainly when they are interpreted together with ABA-responsive genes, ROS-related enzymes and transcription factors.

Transcription factor families provide the main regulatory connection between signaling and downstream genes. WRKY factors are involved in ROS signaling, ABA response, pathogen-related stress and antioxidant regulation [135]. DREB/CBF factors are associated with ABA-independent osmotic, dehydration, cold and salt responses. NAC factors regulate stress adaptation, senescence, ROS-related responses and cell wall remodeling [136]. bZIP factors, especially ABF/AREB-type regulators, are central components of ABA-dependent salt and drought responses [132]. MYB factors participate in phenylpropanoid metabolism, flavonoid biosynthesis, osmotic regulation and cell wall-related pathways [137]. This overlap explains why the same transcription factor families are detected in drought, salinity, temperature, and biotic-stress datasets, although their downstream targets may differ depending on tissue, species, and stress intensity.

Available transcriptomic data support the relevance of this framework for *Salicornia*. In *S. persica*, RNA-seq identified 1595 differentially expressed genes, including transcription factors, protein kinases and transporters; gene network analysis indicated that ABA signaling, calcium signaling and sodium compartmentalization are major components of salinity tolerance [138]. In *S. europaea*, transcriptome profiling showed salt-responsive changes in ion transport, carbohydrate and energy metabolism, stress-response pathways and transcriptional regulation [139,140]. Fan et al. also reported that salinity affected cell wall metabolism and lignin biosynthetic pathways and identified a large set of transcription factors in roots and shoots of *S. europaea* [139]. These data indicate that *Salicornia* salt tolerance is controlled by interconnected modules involving ion transporters, antioxidant enzymes, kinase signaling and transcription factors.

For processing-oriented marker discovery, the most useful candidates are therefore not individual stress-induced genes, but coordinated molecular blocks. One block may include ion transport and compartmentalization genes such as SOS1, NHX, HKT and V-ATPase-related genes. A second block may include antioxidant genes such as SOD, CAT, APX, POD/GPX and ascorbate-glutathione cycle genes. A third block may include ABA/Ca^2+^/MAPK signaling components and transcription factors such as WRKY, DREB/CBF, NAC, bZIP and MYB. When these blocks are correlated with residual salt content, water retention, oxidative stability, color preservation, tissue firmness, and extractability, they become more biologically meaningful than single-gene markers.

**Figure 4:**
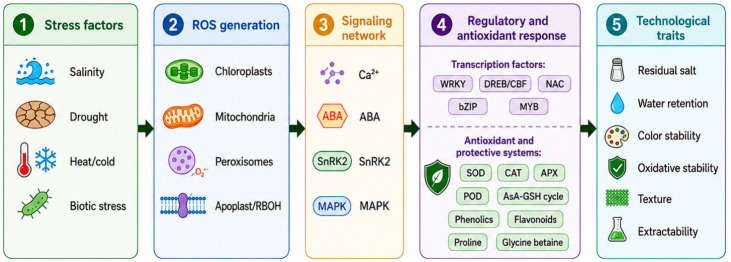
ROS generation and antioxidant defense network under salinity and related stress responses in *Salicornia*.

The ROS-centered mechanism linking salinity perception, stress signaling, antioxidant defense, and processing-related biomass traits is summarized in Figure 4.

## 8. *Salicornia* as a Model Halophytic Raw Material for Processing-Oriented Transcriptomic Markers

*Salicornia* is a specific type of plant raw material because its processability characteristics are formed under constant salinity pressure. Therefore, transcriptomic markers for this crop should not be selected only as general stress markers. They should be linked to practical processing traits, including residual salinity, water retention, tissue texture, drying stability, extractability of soluble fractions, oxidative stability, and sensory quality. In this context, *Salicornia* is not only a model of salt tolerance, but also a model for linking salinity-regulated pathways with the technological behavior of halophytic biomass [141,142].

The marker groups listed in Table 1 should be considered candidate modules rather than validated diagnostic markers. Experimental validation should include at least four levels: targeted confirmation of transcript abundance by qPCR, biochemical validation by enzyme or metabolite assays, structural or ion validation by cell-wall analysis and ion profiling, and direct comparison with processability measurements. For *Salicornia*, the most relevant validation traits are residual salt content, drying loss, rehydration capacity, tissue firmness, color stability, oxidative stability, and extract yield. A marker should be considered practically useful only when its expression remains stable across species or ecotypes, salinity levels, developmental stages and independent batches.

### Dataset-Informed Synthesis of Candidate Marker Modules

The marker panel proposed in this review is based on a qualitative synthesis of available *Salicornia* transcriptomic, biochemical and omics studies rather than on a new quantitative RNA-seq meta-analysis. A formal cross-dataset meta-analysis is currently limited by differences in species, tissue type, salinity level, treatment duration, developmental stage, sequencing strategy, reference annotation, and metadata completeness. These limitations are important because a transcript that is strongly induced in one experiment may reflect a species-specific or tissue-specific response rather than a transferable marker for biomass processability.

Despite these limitations, several recurrent modules can be extracted from the available evidence. Transcriptomic studies of *S. europaea* have shown salt-responsive regulation of ion transport, osmotic adjustment, carbohydrate and energy metabolism, stress-response pathways, and transcription factors [139,140]. In *S. persica*, RNA-seq profiling identified differentially expressed genes associated with ABA signaling, calcium regulation, transporters and sodium compartmentalization [138]. Comparative biochemical analysis of *S. persica*, *S. europaea* and *S. bigelovii* further showed species-dependent antioxidant and osmoprotective responses, including changes in SOD, POD, APX, proline and glycine betaine [129]. In addition, transcriptomic-lipidomic work in *S. europaea* supports the importance of lipid remodeling, including phospholipid and sphingolipid-related pathways [57], while cell-wall studies showed salinity-dependent changes in cellulose deposition, pectin methylesterification, lignin composition and wall stiffness [143,144] (Table 2).

Therefore, the marker groups proposed here should be interpreted as a dataset-informed candidate framework. The strongest cross-study candidates are not individual transcripts, but functional modules related to ion transport and vacuolar compartmentalization, osmoprotection, antioxidant defense, cell wall remodeling and lipid metabolism. Future integrative analysis should harmonize public RNA-seq datasets by species, tissue, salinity level, exposure time and annotation version, followed by re-analysis using a common pipeline. Only after such harmonization should compact qPCR panels be finalized for processability prediction.

The gene names listed below should be interpreted as candidate genes or gene families for marker development. *Salicornia*-specific gene names are used where they have been reported in published studies, whereas conserved gene-family names are used when exact species-specific orthology still requires annotation and validation.

The first important marker group is related to ion transport and vacuolar compartmentalization. Fan et al. and Ma et al. showed that salt treatment in *Salicornia europaea* activates transcripts associated with ion transport, osmotic regulation and stress adaptation [139,140]. Lv et al. demonstrated that the V ATPase subunit A participates in salt tolerance through vacuolar Na^+^ compartmentalization [146]. For processing, this pathway can be interpreted as follows: activation of tonoplast proton pumps and ion transport systems increases ion sequestration in vacuoles, supports cellular osmotic balance, and changes tissue hydration. Therefore, transcripts related to V ATPase activity, vacuolar ion compartmentalization and Na^+^ redistribution may be considered candidate markers for residual salinity, water retention and dehydration behavior of *Salicornia* biomass.

The second marker group is linked to osmoprotection. Under salt stress, *Salicornia* activates pathways associated with osmolyte accumulation, including proline and glycine betaine [140]. These metabolites help maintain cell turgor and protect proteins and membranes under osmotic stress. From a technological perspective, this pathway is important because it may influence drying stability, rehydration capacity, and texture after processing. The marker chain can be written as follows: salinity signal, activation of osmoprotective genes, osmolyte accumulation, stabilization of cellular water status, and improved resistance to dehydration. Therefore, osmoprotection-related transcripts can be used as candidate indicators for predicting drying behavior and water-holding capacity.

The third marker group comprises antioxidant defense and stress signaling. In *Salicornia*, this regulatory layer is relevant not only to salt tolerance but also to raw material quality, as oxidative stress affects membrane stability, pigments, proteins, and bioactive metabolites. Candidate markers in this group may include SOD, CAT, APX, POD/GPX, ascorbate-glutathione cycle genes, and regulatory genes from the WRKY, DREB/CBF, NAC, bZIP, and MYB families. Their technological relevance is linked to oxidative stability, color preservation, storage resistance, and the stability of functional compounds during drying and storage.

The fourth and probably most relevant group for processing is cell wall remodeling. Cardenas Perez et al. showed that *Salicornia* ecotypes differ in cell wall remodeling strategies [143]. Perez et al. further demonstrated that salinity changes cell wall nanomechanics and lignin composition in *Salicornia* [144]. This is directly related to processing quality. Cell wall stiffness, lignification and matrix organization determine tissue firmness, grindability, water release and extractability of cell wall associated compounds. The pathway can be explained as follows: salinity changes expression of cell wall remodeling genes, this modifies lignin and wall mechanics, then biomass texture, milling behavior and extraction efficiency change. Therefore, transcripts linked with lignin biosynthesis, cell wall loosening, pectin modification and structural polysaccharide remodeling are strong candidate markers for texture, firmness and extractability.

The fifth marker group is lipid metabolism. Yang et al. showed that lipid metabolism contributes to salt tolerance in *Salicornia europaea* [57]. This is important for processing because lipid remodeling can affect membrane stability, oxidative resistance and the nutritional profile of biomass. If *Salicornia* is used as a functional ingredient, changes in lipid-related transcripts may help predict oxidative stability and the value of lipid-containing fractions. The pathway can be described as follows: salinity regulated lipid metabolism, change in membrane and fatty acid composition, improved membrane stability, altered oxidative stability and nutritional quality.

A separate factor is the microbiota. Mesa et al. described endophytic bacteria of *Salicornia ramosissima* with plant growth promoting potential [147], while studies on halophyte systems show that salinity can change microbial communities and influence plant stress responses [49]. This means that some transcriptomic signatures in *Salicornia* may reflect not only plant genotype and salinity level, but also plant microbiome interactions. For marker development, this is important because samples from different sites may show different expression profiles even under similar salinity. Therefore, soil conditions and microbial context should be recorded when comparing *Salicornia* populations or production sites.

For practical use, *Salicornia* markers should be organized as a compact multi-pathway panel rather than as single genes. A useful panel may include ion transport and vacuolar compartmentalization markers for residual salinity and water retention, osmoprotection markers for dehydration tolerance, SOD, APX, POD, and non-enzymatic antioxidant markers for oxidative stability, color preservation, and storage resistance, cell wall remodeling markers for texture and extractability, and lipid metabolism markers for oxidative stability and nutritional quality. Such a panel should be validated together with direct processing measurements, including moisture loss during drying, rehydration capacity, tissue firmness, grinding behavior, extract yield, residual salt content and color stability. In this form, transcriptomic analysis can move *Salicornia* research from a general description of salt tolerance to practical prediction of biomass quality for processing. However, before this panel can be used as a diagnostic tool, it should be tested through harmonized re-analysis of public RNA-seq datasets and validated against independent processability measurements.

## 9. Emerging Genomic and AI-Assisted Approaches for *Salicornia* Quality Prediction

Genomic and biotechnological approaches can strengthen transcriptomic marker discovery in *Salicornia* by separating correlative expression responses from functionally relevant genes. Current transcriptomic studies have already identified salt-responsive candidates related to ion transport, osmotic adjustment, antioxidant defense, lipid metabolism, cell wall remodeling, and transcriptional regulation. However, most of these candidates still require validation beyond differential expression. Therefore, RNA-seq-based marker discovery should be combined with functional genomics, population-level association analysis and multi-omics phenotyping.

CRISPR/Cas genome editing provides a direct strategy for testing whether candidate genes are truly involved in salt tolerance and biomass-quality traits. Direct CRISPR examples in *Salicornia* remain limited, and efficient transformation and regeneration systems remain a practical bottleneck for many halophytic species. Therefore, evidence from major crops can be used as a methodological reference. In rice, CRISPR/Cas9 editing of OsRR22 and OsDSG1 improved salinity tolerance, while editing of GmAITR genes enhanced salt tolerance in soybean [148,149,150]. For *Salicornia*, priority targets may include ion transport and compartmentalization genes such as SeHKT1;2, SeSOS1, SeNHX1, SeVHA-A and SeVP1, antioxidant genes such as SOD, CAT, APX and POD, and regulatory genes from WRKY, DREB/CBF, NAC, bZIP and MYB families.

GWAS, TWAS, and genomic selection can complement transcriptomics by linking natural genetic variation with salinity-related and processability traits. In rice, GWAS identified OsWRKY53 as a regulator of salt tolerance connected with OsMKK10.2 and OsHKT1;5, while barley GWAS identified salt-tolerance associations near the HKT1;5 region [151,152]. For *Salicornia*, similar approaches would require panels of species, ecotypes or populations grown under controlled salinity, followed by parallel measurement of genotype, transcript abundance, ion accumulation, growth, succulence and processing traits. This is important because *Salicornia* biomass quality is likely to depend on genotype, salinity regime, developmental stage and tissue type rather than on one universal marker.

Multi-omics integration is necessary because transcript abundance alone does not confirm protein activity, metabolite accumulation, or processability performance. In *Salicornia*, the clearest metabolomic example is the widely targeted metabolomic analysis of S. europaea under different NaCl treatments. This study detected 552 metabolites, of which 500 were differentially accumulated under salinity. The responsive metabolites included lipids, organic acids, saccharides, alcohols, amino acids, flavonoids, phenolic acids and alkaloids. Importantly, proline, sucrose, glucose, quercetin derivatives and kaempferol derivatives were identified among salt-responsive metabolites, while glycolysis, flavone/flavonol biosynthesis and phenylpropanoid biosynthesis were highlighted as key pathways involved in osmotic tolerance and antioxidant activity [153]. This metabolomic evidence agrees with transcriptomic data showing that salinity affects osmotic adjustment, primary metabolism, antioxidant defense and secondary metabolism in S. europaea [139,140].

A more direct example of multi-omics integration is transcriptomic-lipidomic analysis of *S. europaea* under NaCl treatment. This study showed that salt adaptation was associated with lipid remodeling, especially changes in phospholipid and sphingolipid-related pathways, and reported salt-responsive expression of genes such as SeSOS1, SeNHX1, SeVHA-A, SeVP1 and SePSS [57]. These results are consistent with transcriptomic evidence for ion transport and vacuolar compartmentalization, but they add an additional phenotype-level layer by showing that membrane lipid composition is also reorganized under salinity. For processability prediction, this connection is important because lipid remodeling may affect membrane stability, oxidative resistance, drying tolerance, and the functional value of lipid-containing fractions.

Proteomic evidence in *Salicornia* is more limited than transcriptomic and metabolomic evidence, but it is still useful for addressing the mechanism-phenotype gap. Comparative proteomic analysis of *S. europaea* shoots under different salinity levels provided proteome reference maps and detected salt-responsive protein spots, indicating that salinity affects proteins involved in photosynthesis, energy metabolism, stress response, and antioxidant protection. This partially supports transcriptomic interpretations of salt-induced metabolic adjustment, but it also shows why transcript abundance cannot be treated as direct evidence of protein activity. Therefore, proposed *Salicornia* markers should be evaluated through combined transcriptomics, proteomics, metabolomics, lipidomics, ionomics and cell-wall phenotyping. AI-assisted models can then rank genes, proteins, metabolites and modules associated with residual salinity, drying loss, rehydration capacity, color stability, texture and extract yield, but such models should be externally validated and interpreted through known biological pathways rather than used as black-box predictors.

## 10. Conclusions

Transcriptomic data are most informative in studies of plant raw material quality not as a substitute for technological testing, but as a mechanistic layer that links variation in raw materials, cultivation and storage conditions, and pretreatment effects to the regulation of pathways controlling the synthesis, deposition, and remodeling of key biopolymers. In this context, transcriptomics makes the processing phenotype biologically interpretable and supports a shift from descriptive quality assessment toward mechanistically grounded prediction of processing behavior.

For most processability traits, network-level markers, including pathway signatures and co-expression modules, are more robust and transferable than single genes because they better capture the polygenic and context-dependent architecture of quality formation. Their practical value is greatest when translated into compact and interpretable marker panels suitable for targeted quality control assays. At the same time, causal interpretation of regulatory nodes requires orthogonal validation using genetic, functional, proteomic, and metabolomic evidence. The *Salicornia* case further demonstrates that in halophytic raw materials, processability traits are strongly shaped by stress-induced transcriptomic programs, particularly those associated with salinity, ion homeostasis, osmoregulation, and cell wall remodeling. Accordingly, reliable marker development for *Salicornia* should rely on modular signatures, strict standardization of salinity conditions and developmental stages, and independent validation across seasons and sites. A priority for future work is the harmonized re-analysis of publicly available *Salicornia* transcriptomic datasets using a common annotation and statistical pipeline, followed by qPCR and multi-omics validation of the most stable marker modules.

## Figures and Tables

**Table 1:** Processing-related biomass traits and candidate molecular modules in *Salicornia* spp.

Processability Trait	Measurable Phenotype	Candidate Genes, Gene Families or Pathways	Supporting Evidence and Validation Need
Residual salt content	Na^+^, K^+^, Na^+^/K^+^ ratio, ash content	SeHKT1;2, SeSOS1, SeNHX1, SeVHA-A, SeVP1, HKT, SOS1, NHX, V-ATPase	Transcriptomic studies in *S. europaea* and *S. persica* indicate activation of ion transport and vacuolar compartmentalization pathways under salinity. Validation requires ionomic analysis and comparison with residual salt after drying or processing.
Water retention and drying behavior	Moisture loss, dehydration rate, rehydration capacity	P5CS, P5CR, BADH, CMO, SPS, SUS, PIP, TIP, proline and glycine betaine pathways	Osmolyte accumulation is repeatedly associated with salinity adaptation in *Salicornia*. Validation requires parallel measurement of osmolytes, water activity, drying loss and rehydration index.
Oxidative and color stability	MDA, H_2_O_2_, pigment retention, browning or color change during storage	SOD, CAT, APX, POD/GPX, GR, MDHAR, DHAR, phenylpropanoid/flavonoid genes	Comparative studies show species-dependent antioxidant responses in *S. persica*, *S. europaea* and *S. bigelovii*. Validation requires enzyme assays, metabolite profiling and color-stability testing.
Texture and tissue firmness	Firmness, grindability, particle-size behavior, mechanical resistance	CESA, PME, PMEI, EXP, XTH, PAL, C4H, 4CL, CCR, CAD, POD/laccase-related genes	Cell-wall studies in *S. europaea* show that salinity can alter cellulose deposition, pectin methylesterification, lignin composition and wall stiffness. Validation requires cell-wall chemistry and mechanical phenotyping.
Extractability of soluble and functional fractions	Extract yield, soluble solids, phenolic and flavonoid recovery	PME, PMEI, EXP, XTH, cellulase/hemicellulase-related genes, PAL, CHS, FLS, flavonoid pathway genes	Extractability is expected to depend on cell-wall structure and secondary metabolite accumulation. Validation requires extraction assays, metabolomics and compound-level profiling.
Lipid-related stability and functional quality	Lipid composition, membrane stability, oxidation susceptibility	SePSS, KAS, FAD, GPAT, LACS, PLD, LOX, phospholipid and sphingolipid metabolism genes	Transcriptomic and lipidomic studies in *S. europaea* show salt-induced lipid remodeling. Validation requires lipidomics and oxidative-stability measurements.

**Table 2:** Dataset-informed evidence supporting candidate marker modules in *Salicornia* spp.

Source of Evidence	Species and Material	Main Stress or Omics Context	Recurrent Modules Relevant to Marker Development	Use for the Proposed Panel
Ma et al. [139]	*S. europaea*, shoots	NaCl-responsive transcriptome	Ion transport, stress-response pathways, carbohydrate and energy metabolism	Supports inclusion of ion transport, osmotic adjustment and stress-response modules
Fan et al. [140]	*S. europaea*, roots and shoots	Salinity-responsive transcriptome	Primary metabolism, transcription factors, cell wall metabolism, lignin biosynthesis	Supports inclusion of TF modules and cell-wall remodeling markers
Aliakbari et al. [138]	*S. persica*	RNA-seq under salinity	ABA signaling, calcium regulation, transporters, sodium compartmentalization	Supports inclusion of ABA/Ca^2+^ signaling and ion compartmentalization markers
Homayouni et al. [129]	*S. persica*, *S. europaea*, *S. bigelovii*	Comparative biochemical response to 300 and 500 mM NaCl	SOD, POD, APX, proline, glycine betaine, ion accumulation	Supports antioxidant and osmoprotective modules and shows species-dependent efficiency
Yang et al. [57]	*S. europaea*	Transcriptomic and lipidomic response to NaCl	Lipid biosynthesis, phospholipid and sphingolipid remodeling, membrane-related pathways	Supports lipid metabolism markers for membrane stability and oxidative resistance
Cárdenas Pérez et al. [143]	*S. europaea* ecotypes	Cell-wall response under salinity	Cellulose deposition, pectin methylesterification, lignin composition, wall stiffness	Supports cell-wall remodeling markers for texture and extractability
Wang et al. [145]	*S. europaea*, shoots	Comparative proteomic response under different salinity levels	Photosynthesis, energy metabolism, stress-response proteins, antioxidant-related proteins	Supports proteomic validation of transcriptomic modules and shows that transcript abundance should not be treated as protein-level proof
Duan et al. [138]	*S. europaea*	Widely targeted metabolomics under NaCl treatments	Proline, sucrose, glucose, quercetin derivatives, kaempferol derivatives, flavone/flavonol and phenylpropanoid pathways	Supports osmoprotection, antioxidant and secondary-metabolism modules relevant to nutritional and processability traits

## Data Availability

No new data were created or analyzed in this study. Data sharing is not applicable to this article.

## References

[1] Zhu J.K. (2016). Abiotic stress signaling and responses in plants. Cell.

[2] Munns R., Tester M. (2008). Mechanisms of salinity tolerance. Annu. Rev. Plant Biol..

[3] Zhao S., Cao R., Sun L., Zhuang D., Zhong M., Zhao F., Jiao G., Chen P., Li X., Duan Y. (2024). An Integrative Analysis of the Transcriptome and Proteome of Rice Grain Chalkiness Formation Under High Temperature. Plants.

[4] Nirmal R.C., Furtado A., Wrigley C., Henry R.J. (2016). Influence of gene expression on hardness in wheat. PLoS ONE.

[5] De Angelis D., Latrofa V., Squeo G., Pasqualone A., Summo C. (2024). Techno-functional, rheological, and chemical properties of plant-based protein ingredients obtained with dry fractionation and wet extraction. Curr. Res. Food Sci..

[6] Gan J., Qiu Y., Tao Y., Zhang L., Okita T.W., Yan Y., Tian L. (2024). RNA-seq analysis reveals transcriptome reprogramming and alternative splicing during early response to salt stress in tomato root. Front. Plant Sci..

[7] Kirwan J.A., Gika H., Beger R.D., Bearden D., Dunn W.B., Goodacre R., Theodoridis G., Witting M., Yu L.R., Wilson I.D. (2022). Quality assurance and quality control reporting in untargeted metabolic phenotyping: mQACC recommendations for analytical quality management. Metabolomics.

[8] Faqeerzada M.A., Park E., Lim J., Kim K., Sathasivam R., Park S.U., Kim H., Cho B.K. (2025). Development of multi-sensing technologies for high-throughput morphological, physiological, and biochemical phenotyping of drought-stressed watermelon plants. Plant Physiol. Biochem..

[9] Berlingeri J., Fuentes A., Ranario E., Yun H., Rim E.Y., Garrett O., Howard A., LaPorte M.F., Lo S., Pauli D. (2025). Integration of crop modeling and sensing into molecular breeding for nutritional quality and stress tolerance. Theor. Appl. Genet..

[10] Upton R.N., Correr F.H., Lile J., Reynolds G.L., Falaschi K., Cook J.P., Lachowiec J. (2023). Design, execution, and interpretation of plant RNA seq analyses. Front. Plant Sci..

[11] Butardo V.M., Luo J., Li Z., Gidley M.J., Bird A.R., Tetlow I.J., Fitzgerald M., Jobling S.A., Rahman S. (2020). Functional genomic validation of the roles of *soluble starch synthase IIa* in *Japonica* rice endosperm. Front. Genet..

[12] Li Y., Li P., Zhao D., Wang F., Lu B. (2025). Metabolic and starch biosynthetic networks underlying yam waxiness formation revealed by integrated transcriptomics and proteomics. J. Agric. Food Chem..

[13] Song J., Ma C., Xu Y., Wang Y., Wang B., Zhang G., Liu X., Xu X., Yang Y., Zhang N. (2025). Mechanistic analysis of starch gelatinization properties regulated by biomolecules: A protein, non-starch polysaccharides and lipids perspective. Crit. Rev. Food Sci. Nutr..

[14] Vogel C., Marcotte E.M. (2012). Insights into the regulation of protein abundance from proteomic and transcriptomic analyses. Nat. Rev. Genet..

[15] Jensen O.N. (2006). Interpreting the protein language using proteomics. Nat. Rev. Mol. Cell Biol..

[16] Moskal K., Puchta-Jasińska M., Bolc P., Motor A., Frankowski R., Pietrusińska-Radzio A., Rucińska A., Tomiczak K., Boczkowska M. (2025). Why “Where” Matters as Much as “How Much”: Single-Cell and Spatial Transcriptomics in Plants. Int. J. Mol. Sci..

[17] Panditrao G., Bhowmick R., Meena C., Sarkar R.R. (2022). Emerging landscape of molecular interaction networks: Opportunities, challenges and prospects. J. Biosci..

[18] Chen Y., Zhu H., Feng Y., Wang Y., Wang X., Zhang W., Luo Y. (2026). Rheological analysis in food processing: Factors, applications, and future outlooks with machine learning integration. RSC Adv..

[19] Malpartida Yapias R.J., Ore Areche F., Echevarria Victorio J.P., Paucarchuco Soto J., Lobato Calderon G.R., Palomino Santos E.R., Montalvo Otivo J.M., Flores-Miranda C., Ruiz Rodriguez A. (2025). Advancements in green extraction technologies for pectin enhancing efficiency, sustainability, and functional properties: A systematic review. Braz. J. Biol..

[20] Spinardi A., Cola G., Gardana C.S., Mignani I. (2019). Variation of anthocyanin content and profile throughout fruit development and ripening of highbush blueberry cultivars grown at two different altitudes. Front. Plant Sci..

[21] Xie M., Wang J., Wang F., Wang J., Yan Y., Feng K., Chen B. (2025). A review of genomic, transcriptomic, and proteomic applications in edible fungi biology: Current status and future directions. J. Fungi.

[22] Muthuramalingam K., Kim Y., Cho M. (2022). β-glucan, “the knight of health sector”: Critical insights on physiochemical heterogeneities, action mechanisms and health implications. Crit. Rev. Food Sci. Nutr..

[23] Desai N., Rana D., Salave S., Gupta R., Patel P., Karunakaran B., Sharma A., Giri J., Benival D., Kommineni N. (2023). Chitosan: A potential biopolymer in drug delivery and biomedical applications. Pharmaceutics.

[24] Zhang P.P., Li M.S., Zhou J., Zhu C.H., Tang R., He Z.C., Yao X.H., Ping Y.F., Xiang D.F., Tan L.Y. (2026). Region-resolved proteomic map of the human brain: Functional interconnections and neurological implications. Signal Transduct. Target. Ther..

[25] Zhan C., Tang T., Wu E., Zhang Y., He M., Wu R., Bi C., Wang J., Zhang Y., Shen B. (2023). From multi-omics approaches to personalized medicine in myocardial infarction. Front. Cardiovasc. Med..

[26] Huo J., Yu M., Feng N., Zheng D., Zhang R., Xue Y., Khan A., Zhou H., Mei W., Du X. (2024). Integrated transcriptome and metabolome analysis of salinity tolerance in response to foliar application of choline chloride in rice (*Oryza sativa* L.). Front. Plant Sci..

[27] Satrio R.D., Fendiyanto M.H., Miftahudin M. (2024). Tools and techniques used at global scale through genomics, transcriptomics, proteomics, and metabolomics to investigate plant stress responses at the molecular level. Molecular Dynamics of Plant Stress and Its Management.

[28] Hernández-Elvira M., Sunnerhagen P. (2022). Post-transcriptional regulation during stress. FEMS Yeast Res..

[29] Lin Y., Lin P., Lu Y., Zheng J., Zheng Y., Huang X., Zhao X., Cui L. (2024). Post-translational modifications of RNA-modifying proteins in cellular dynamics and disease progression. Adv. Sci..

[30] Dal Co A., Ackermann M., van Vliet S. (2023). Spatial self-organization of metabolism in microbial systems: A matter of enzymes and chemicals. Cell Syst..

[31] Wang P., Moreno S., Janke A., Boye S., Wang D., Schwarz S., Voit B., Appelhans D. (2022). Probing crowdedness of artificial organelles by clustering polymersomes for spatially controlled and pH-triggered enzymatic reactions. Biomacromolecules.

[32] Tardy B.L., Mattos B.D., Otoni C.G., Beaumont M., Majoinen J., Kamarainen T., Rojas O.J. (2021). Deconstruction and reassembly of renewable polymers and biocolloids into next generation structured materials. Chem. Rev..

[33] Arrigo R., Malucelli G., Mantia F.P.L. (2021). Effect of the elongational flow on the morphology and properties of polymer systems: A brief review. Polymers.

[34] Oyinloye T.M., Lee C.J., Yoon W.B. (2025). Integration of nonlinear rheology and CFD simulation to elucidate the influence of saturated oil on soy protein concentrate behavior during high-moisture extrusion. Gels.

[35] Bhaskar P.B., Wu L., Busse J.S., Whitty B.R., Hamernik A.J., Jansky S.H., Buell C.R., Bethke P.C., Jiang J. (2010). Suppression of the vacuolar invertase gene prevents cold-induced sweetening in potato. Plant Physiol..

[36] Liu T., Zhou T., Cui G., Liu X., Song B. (2025). Advances in understanding cold-induced sweetening in potato tubers. Hortic. Plant J..

[37] Wang Q., Li Y., Sun F., Li X., Wang P., Yang G., He G. (2019). Expression of *Puroindoline* a in durum wheat affects milling and pasting properties. Front. Plant Sci..

[38] Panahi B., Hamid R., Ghorbanzadeh Z., Golkari S., Yildirim M., Jacob F. (2025). Transcriptional networks shaping malting quality in barley: From grain development to brewing performance. Food Chem. Mol. Sci..

[39] Held S.T. (2022). Effects of Mash Style on β-Glucan Concentration and β-Glucanase Activity in the Production of Quality Wort for Beer Brewing. Master’s Thesis.

[40] Fox G.P. (2025). Variation in Barley Quality and Its Impact on Malting and Brewing. Ph.D. Thesis.

[41] Amirebrahimi F.F., Saidi A., Ahmadikhah A. (2025). Genome-wide association study (GWAS) and transcription analysis of candidate genes for rice grain eating and cooking quality traits. BMC Genom..

[42] Zhao Y., Zhao J., Hu M., Sun L., Liu Q., Zhang Y., Li Q., Wang P., Ma W., Li H. (2023). Transcriptome and proteome analysis revealed the influence of high-molecular-weight glutenin subunits deficiency on expression of storage substances and the potential regulatory mechanism of HMW-GSs. Foods.

[43] Yang T., Wang B., Lv T., Wang P., Zhou Q., Jiang D., Jiang H. (2025). Investigating the molecular mechanism of high-molecular-weight glutenin subunit affects gluten aggregation during dough mixing: Experimental characterizations and computational simulations. Food Chem..

[44] Liu S., Xie L., Wu H., Xu D., Che R., Tian W., Liu B., Chao Y., Zhang Y., Xia X. (2025). TaIAA10-6D orchestrates processing quality and grain yield by modulating glutenin–gliadin ratio and plant morphogenesis in wheat. Crop J..

[45] Xie L., Liu S., Zhang Y., Tian W., Xu D., Li J., Luo X., Li L., Bian Y., Li F. (2023). Efficient proteome-wide identification of transcription factors targeting Glu-1: A case study for functional validation of TaB3-2A1 in wheat. Plant Biotechnol. J..

[46] Merlino M., Gaudin J.C., Dardevet M., Martre P., Ravel C., Boudet J. (2023). Wheat DOF transcription factors TaSAD and WPBF regulate glutenin gene expression in cooperation with SPA. PLoS ONE.

[47] Li C., Mur L.A., Wang Q., Hou X., Zhao C., Chen Z., Wu J., Guo Q. (2022). ROS scavenging and ion homeostasis is required for the adaptation of halophyte *Karelinia caspia* to high salinity. Front. Plant Sci..

[48] Wang X., Wang T., Yu P., Li Y., Lv X. (2024). NO enhances the adaptability to high-salt environments by regulating osmotic balance, antioxidant defense, and ion homeostasis in eelgrass based on transcriptome and metabolome analysis. Front. Plant Sci..

[49] Yu Y., Wang H., Liu J., Wang Q., Shen T., Guo W., Wang R. (2012). Shifts in microbial community function and structure along the successional gradient of coastal wetlands in Yellow River Estuary. Eur. J. Soil Biol..

[50] Marand A.P., Eveland A.L., Kaufmann K., Springer N.M. (2023). cis-Regulatory elements in plant development, adaptation, and evolution. Annu. Rev. Plant Biol..

[51] Kulkarni S.R., Vaneechoutte D., Van de Velde J., Vandepoele K. (2018). TF2Network: Predicting transcription factor regulators and gene regulatory networks in *Arabidopsis* using publicly available binding site information. Nucleic Acids Res..

[52] Song X., Li Y., Cao X., Qi Y. (2019). MicroRNAs and their regulatory roles in plant–environment interactions. Annu. Rev. Plant Biol..

[53] Zeng Z., Zhang S., Li W., Chen B., Li W. (2022). Gene-coexpression network analysis identifies specific modules and hub genes related to cold stress in rice. BMC Genom..

[54] Yu T., Zhang J., Cao J., Ma X., Li W., Yang G. (2023). Hub gene mining and co-expression network construction of low-temperature response in maize of seedling by WGCNA. Genes.

[55] Tian F., Yang D.C., Meng Y.Q., Jin J., Gao G. (2020). PlantRegMap: Charting functional regulatory maps in plants. Nucleic Acids Res..

[56] Hussey S.G., Mizrachi E., Creux N.M., Myburg A.A. (2013). Navigating the transcriptional roadmap regulating plant secondary cell wall deposition. Front. Plant Sci..

[57] Yang L., Wang Y., Bai Y., Yang J., Gao Y., Hou C., Gao M., Gu X., Liu W. (2025). Lipid metabolism improves salt tolerance of *Salicornia europaea*. Ann. Bot..

[58] Cao Y., Ma J., Cheng Z., Xia L., Wang L., Li J., Zhang X., Han S., Tian Y., Li M. (2026). The Zma-miRNA319-ZmMYB74 module regulates maize resistance to stalk rot disease by modulating lignin deposition. Plant Biotechnol. J..

[59] Arshad K.T., Li C., Li L., Wang J., Chen J., Zhao Y. (2025). Genome-wide identification and expression profiling of bHLH transcription factors associated with ferulic acid biosynthesis in *Angelica sinensis*. Front. Plant Sci..

[60] Qiao X., Jian B., Qiu J., Singh J., Kaur I., Hao R., Singh J., Singh L., Zhang H., Yin X. (2025). Transcriptomics in solanaceous crop improvement: Advances and opportunities. Front. Plant Sci..

[61] Zhu Z., Jiang L., Ding X. (2023). Advancing breast cancer heterogeneity analysis: Insights from genomics, transcriptomics and proteomics at bulk and single-cell levels. Cancers.

[62] Yao J., Marand A.P., Bai Y., Schmitz R.J., Fan L. (2025). Advances in plant spatial multi-omics data analysis. Trends Plant Sci..

[63] Zhan J., Meyers B.C. (2023). Plant small RNAs: Their biogenesis, regulatory roles, and functions. Annu. Rev. Plant Biol..

[64] Chen S., Li Y., Hu J., Li H., Hu C., Zhao J., Qian H., Bai S., Tang Z., Feng Y. (2025). Integrating bulk RNA-seq, scRNA-seq and spatial transcriptomics data to identify novel post-translational modification-related molecular subtypes and therapeutic responses in hepatocellular carcinoma. Cancer Cell Int..

[65] Littman R., Cheng M., Wang N., Peng C., Yang X. (2023). SCING: Inference of robust, interpretable gene regulatory networks from single-cell and spatial transcriptomics. iScience.

[66] Wu Z., Sun Y., Zhao X., Liu Z., Zhou W., Niu Y. (2024). Phenotype prediction in plants is improved by integrating large-scale transcriptomic datasets. NAR Genom. Bioinform..

[67] Yang X.S., Wu J., Ziegler T.E., Yang X., Zayed A., Rajani M.S., Zhou D., Basra A.S., Schachtman D.P., Peng M. (2011). Gene expression biomarkers provide sensitive indicators of in planta nitrogen status in maize. Plant Physiol..

[68] Lu S., Sturtevant D., Aziz M., Jin C., Li Q., Chapman K.D., Guo L. (2018). Spatial analysis of lipid metabolites and expressed genes reveals tissue-specific heterogeneity of lipid metabolism in high- and low-oil *Brassica napus* seeds. Plant J..

[69] Knoch D., Rugen N., Thiel J., Heuermann M.C., Kuhlmann M., Rizzo P., Meyer R.C., Wagner S., Schippers J.H.M., Braun H.-P. (2025). A spatio-temporal transcriptomic and proteomic dataset of developing *Brassica napus* seeds. Sci. Data.

[70] Wang J., Fu D., Tian Y., Lv M., Xu J., Yu T., Lu L., Pang X., Li X. (2025). Single-cell transcriptome sequencing reveals dynamic gene expression trajectories regulating vascular cell senescence in *Hylocereus undatus*. Gene.

[71] Yao Y., Xiong E., Qu X., Li J., Liu H., Quan L., Lu W., Zhu X., Chen M., Li K. (2023). WGCNA and transcriptome profiling reveal hub genes for key development stage seed size/oil content between wild and cultivated soybean. BMC Genom..

[72] Jia K., Shen J. (2024). Transcriptome-wide association studies associated with Crohn’s disease: Challenges and perspectives. Cell Biosci..

[73] Yasir M., Kanwal H.H., Hussain Q., Riaz M.W., Sajjad M., Rong J., Jiang Y. (2022). Status and prospects of genome-wide association studies in cotton. Front. Plant Sci..

[74] Benitez A., Iglesias-Moya J., Segura M., Carvajal F., Palma F., Garrido D., Martínez C., Jamilena M. (2022). RNA-seq based analysis of transcriptomic changes associated with ABA-induced postharvest cold tolerance in zucchini fruit. Postharvest Biol. Technol..

[75] Leek J.T., Scharpf R.B., Bravo H.C., Simcha D., Langmead B., Johnson W.E., Geman D., Baggerly K., Irizarry R.A. (2010). Tackling the widespread and critical impact of batch effects in high-throughput data. Nat. Rev. Genet..

[76] Schurch N.J., Schofield P., Gierliński M., Cole C., Sherstnev A., Singh V., Wrobel N., Gharbi K., Simpson G.G., Owen-Hughes T. (2016). How many biological replicates are needed in an RNA-seq experiment and which differential expression tool should you use?. RNA.

[77] Liu Y., Zhou J., White K.P. (2014). RNA-seq differential expression studies: More sequence or more replication?. Bioinformatics.

[78] Rubio-Piña J.A., Zapata-Pérez O. (2011). Isolation of total RNA from tissues rich in polyphenols and polysaccharides of mangrove plants. Electron. J. Biotechnol..

[79] Iqbal A., Yang Y., Wu Y., Li J., Hamayun M., Hussain A., Shah F. (2020). An easy and robust method for the isolation of high quality RNA from coconut tissues. Electron. J. Biotechnol..

[80] Mishko A., Sundyreva M., Stepanov I., Efimenko S., Plotnikov V., Nenko N. (2021). Isolation of high-quality RNA from plant seeds. Biol. Commun..

[81] Ewels P., Magnusson M., Lundin S., Käller M. (2016). MultiQC: Summarize analysis results for multiple tools and samples in a single report. Bioinformatics.

[82] Dobin A., Davis C.A., Schlesinger F., Drenkow J., Zaleski C., Jha S., Batut P., Chaisson M., Gingeras T.R. (2013). STAR: Ultrafast universal RNA-seq aligner. Bioinformatics.

[83] Bray N.L., Pimentel H., Melsted P., Pachter L. (2016). Near-optimal probabilistic RNA-seq quantification. Nat. Biotechnol..

[84] Soneson C., Love M.I., Robinson M.D. (2016). Differential analyses for RNA-seq: Transcript-level estimates improve gene-level inferences. F1000Research.

[85] Zhao L., Zhang H., Kohnen M.V., Prasad K.V., Gu L., Reddy A.S. (2019). Analysis of transcriptome and epitranscriptome in plants using PacBio Iso-Seq and nanopore-based direct RNA sequencing. Front. Genet..

[86] Liao Y., Smyth G.K., Shi W. (2014). featureCounts: An efficient general-purpose program for assigning sequence reads to genomic features. Bioinformatics.

[87] Robinson M.D., Oshlack A. (2010). A scaling normalization method for differential expression analysis of RNA-seq data. Genome Biol..

[88] Conesa A., Madrigal P., Tarazona S., GomezCabrero D., Cervera A., McPherson A., Szcześniak M.W., Gaffney D.J., Elo L.L., Zhang X. (2016). A survey of best practices for RNA-seq data analysis. Genome Biol..

[89] Wagner G.P., Kin K., Lynch V.J. (2012). Measurement of mRNA abundance using RNA-seq data: RPKM measure is inconsistent among samples. Theory Biosci..

[90] Love M.I., Huber W., Anders S. (2014). Moderated estimation of fold change and dispersion for RNA-seq data with DESeq2. Genome Biol..

[91] The Gene Ontology Consortium (2021). The Gene Ontology resource: Enriching a GOld mine. Nucleic Acids Res..

[92] Kanehisa M., Goto S. (2000). KEGG: Kyoto encyclopedia of genes and genomes. Nucleic Acids Res..

[93] Schwacke R., Ponce-Soto G.Y., Krause K., Bolger A.M., Arsova B., Hallab A., Gruden K., Stitt M., Bolger M.E., Usadel B. (2019). MapMan4: A refined protein classification and annotation framework applicable to multi-omics data analysis. Mol. Plant.

[94] Subramanian A., Tamayo P., Mootha V.K., Mukherjee S., Ebert B.L., Gillette M.A., Paulovich A., Pomeroy S.L., Golub T.R., Lander E.S. (2005). Gene set enrichment analysis: A knowledge-based approach for interpreting genome-wide expression profiles. Proc. Natl. Acad. Sci. USA.

[95] Yu G., Wang L.G., Han Y., He Q.Y. (2012). clusterProfiler: An R package for comparing biological themes among gene clusters. OMICS A J. Integr. Biol..

[96] Prieto-Baños S., Nevers Y., Altenhoff A., Warwick Vesztrocy A., Dessimoz C., Glover N.M. (2025). Annotation matters: The effect of structural gene annotation on orthology inference. Bioinformatics.

[97] Ezoe A., Shirai K., Hanada K. (2021). Degree of functional divergence in duplicates is associated with distinct roles in plant evolution. Mol. Biol. Evol..

[98] Zhao K., Rhee S.Y. (2023). Interpreting omics data with pathway enrichment analysis. Trends Genet..

[99] Zhou W., Soghigian J., Xiang Q.Y. (2022). A new pipeline for removing paralogs in target enrichment data. Syst. Biol..

[100] Ma X., Yan H., Yang J., Liu Y., Li Z., Sheng M., Cao Y., Yu X., Yi X., Xu W. (2022). PlantGSAD: A comprehensive gene set annotation database for plant species. Nucleic Acids Res..

[101] Emms D.M., Kelly S. (2019). OrthoFinder: Phylogenetic orthology inference for comparative genomics. Genome Biol..

[102] Huerta-Cepas J., Szklarczyk D., Heller D., Hernández-Plaza A., Forslund S.K., Cook H., Mende D.R., Letunic I., Rattei T., Jensen L.J. (2019). eggNOG 5.0: A hierarchical, functionally and phylogenetically annotated orthology resource based on 5090 organisms and 2502 viruses. Nucleic Acids Res..

[103] Wilkinson M.D., Dumontier M., Aalbersberg I.J., Appleton G., Axton M., Baak A., Blomberg N., Boiten J.W., da Silva Santos L.B., Bourne P.E. (2016). The FAIR guiding principles for scientific data management and stewardship. Sci. Data.

[104] Clough E., Barrett T. (2016). The gene expression omnibus database. Statistical Genomics: Methods and Protocols.

[105] Deutsch E.W., Bandeira N., Sharma V., Perez-Riverol Y., Carver J.J., Kundu D.J., García-Seisdedos D., Jarnuczak A.F., Hewapathirana S., Pullman B.S. (2020). The Proteo-meXchange consortium in 2020: Enabling big data approaches in proteomics. Nucleic Acids Res..

[106] Tai H.H., Lagüe M., Thomson S., Aurousseau F., Neilson J., Murphy A., Bizimungu B., Davidson C., Deveaux V., Bègue Y. (2020). Tuber transcriptome profiling of eight potato cultivars with different cold-induced sweetening responses. Plant Physiol. Biochem..

[107] Hadish J.A., Hargarten H.L., Zhang H., Mattheis J.P., Honaas L.A., Ficklin S.P. (2024). Towards identification of post-harvest fruit quality transcriptomic markers in Malus domestica. PLoS ONE.

[108] Yi M., Nissley D.V., McCormick F., Stephens R.M. (2020). ssGSEA score-based Ras dependency indexes derived from gene expression data reveal potential Ras addiction mechanisms with possible clinical implications. Sci. Rep..

[109] Azodi C.B., Pardo J., VanBuren R., de Los Campos G., Shiu S.H. (2020). Transcriptome-based prediction of complex traits in maize. Plant Cell.

[110] Torres-Rodríguez J.V., Li D., Schnable J.C. (2025). Evolving best practices for transcriptome-wide association studies accelerate discovery of gene–phenotype links. Curr. Opin. Plant Biol..

[111] Lee Y.K., Jang S., Im J., Koh H.J. (2025). Comprehensive GWAS and transcriptome analysis discovered candidate gene associated with starch pasting properties of temperate japonica rice (*Oryza sativa* L.). Rice.

[112] Yu Y., Mai Y., Zheng Y., Shi L. (2024). Assessing and mitigating batch effects in large-scale omics studies. Genome Biol..

[113] Yu Y., Zhang N., Mai Y., Ren L., Chen Q., Cao Z., Chen Q., Liu Y., Hou W., Yang J. (2023). Correcting batch effects in large-scale multiomics studies using a reference-material-based ratio method. Genome Biol..

[114] Goh W.W.B., Yong C.H., Wong L. (2022). Are batch effects still relevant in the age of big data?. Trends Biotechnol..

[115] Antoszewski K., Chmielewska K., Jagiello K., Puzyn T. (2025). Designing RNA sequencing experiments: A practical guide to reproducible gene expression analysis. Comput. Struct. Biotechnol. J..

[116] Sun H., Li C., Li S., Ma J., Li S., Li X., Gao C., Yang R., Ma N., Yang J. (2024). Identification and validation of stable reference genes for RT-qPCR analyses of *Kobresia littledalei* seedlings. BMC Plant Biol..

[117] Langfelder P., Horvath S. (2008). WGCNA: An R package for weighted correlation network analysis. BMC Bioinform..

[118] O’Malley R.C., Huang S.S.C., Song L., Lewsey M.G., Bartlett A., Nery J.R., Galli M., Gallavotti A., Ecker J.R. (2016). Cistrome and epicistrome features shape the regulatory DNA landscape. Cell.

[119] Huynh-Thu V.A., Irrthum A., Wehenkel L., Geurts P. (2010). Inferring regulatory networks from expression data using tree-based methods. PLoS ONE.

[120] Suzuki N., Koussevitzky S., Mittler R., Miller G. (2012). ROS and redox signalling in the response of plants to abiotic stress. Plant Cell Environ..

[121] Baxter A., Mittler R., Suzuki N. (2014). ROS as key players in plant stress signalling. J. Exp. Bot..

[122] Nadarajah K.K. (2020). ROS homeostasis in abiotic stress tolerance in plants. Int. J. Mol. Sci..

[123] Rai G.K., Mishra S., Chouhan R., Mushtaq M., Chowdhary A.A., Rai P.K., Kumar R.R., Kumar P., Perez-Alfocea F., Colla G. (2023). Plant salinity stress, sensing, and its mitigation through WRKY. Front. Plant Sci..

[124] Torres M.A. (2010). ROS in biotic interactions. Physiol. Plant..

[125] Kurusu T., Kuchitsu K., Tada Y. (2015). Plant signaling networks involving Ca^2+^ and Rboh/Nox-mediated ROS production under salinity stress. Front. Plant Sci..

[126] Hasanuzzaman M., Bhuyan M.H.M.B., Zulfiqar F., Raza A., Mohsin S.M., Mahmud J.A., Fujita M., Fotopoulos V. (2020). Reactive oxygen species and antioxidant defense in plants under abiotic stress: Revisiting the crucial role of a universal defense regulator. Antioxidants.

[127] Mishra N., Jiang C., Chen L., Paul A., Chatterjee A., Shen G. (2023). Achieving abiotic stress tolerance in plants through antioxidative defense mechanisms. Front. Plant Sci..

[128] Caverzan A., Passaia G., Rosa S.B., Ribeiro C.W., Lazzarotto F., Margis-Pinheiro M. (2012). Plant responses to stresses: Role of ascorbate peroxidase in the antioxidant protection. Genet. Mol. Biol..

[129] Homayouni H., Razi H., Izadi M., Alemzadeh A., Kazemeini S.A., Niazi A., Vicente O. (2024). Temporal changes in biochemical responses to salt stress in three *Salicornia* species. Plants.

[130] Aghaleh M., Niknam V., Ebrahimzadeh H., Razavi K. (2011). Effect of salt stress on physiological and antioxidative responses in two species of *Salicornia* (*S. persica* and *S. europaea*). Acta Physiol. Plant..

[131] Szymańska K.P., Polkowska-Kowalczyk L., Lichocka M., Maszkowska J., Dobrowolska G. (2019). SNF1-related protein kinases SnRK2.4 and SnRK2.10 modulate ROS homeostasis in plant response to salt stress. Int. J. Mol. Sci..

[132] Hussain Q., Asim M., Zhang R., Khan R., Farooq S., Wu J. (2021). Transcription factors interact with ABA through gene expression and signaling pathways to mitigate drought and salinity stress. Biomolecules.

[133] Golldack D., Li C., Mohan H., Probst N. (2014). Tolerance to drought and salt stress in plants: Unraveling the signaling networks. Front. Plant Sci..

[134] Manna M., Rengasamy B., Sinha A.K. (2023). Revisiting the role of MAPK signalling pathway in plants and its manipulation for crop improvement. Plant Cell Environ..

[135] Khoso M.A., Hussain A., Ritonga F.N., Ali Q., Channa M.M., Alshegaihi R.M., Meng Q., Ali M., Zaman W., Brohi R.D. (2022). WRKY transcription factors (TFs): Molecular switches to regulate drought, temperature, and salinity stresses in plants. Front. Plant Sci..

[136] Xiong H., He H., Chang Y., Miao B., Liu Z., Wang Q., Dong F., Xiong L. (2025). Multiple roles of NAC transcription factors in plant development and stress responses. J. Integr. Plant Biol..

[137] Li J., Han G., Sun C., Sui N. (2019). Research advances of MYB transcription factors in plant stress resistance and breeding. Plant Signal. Behav..

[138] Aliakbari M., Ranjbar G., Nejad G.M. (2021). RNA-seq transcriptome profiling of the halophyte *Salicornia persica* in response to salinity. J. Plant Growth Regul..

[139] Fan P., Nie L., Jiang P., Feng J., Lv S., Chen X., Bao H., Guo J., Tai F., Wang J. (2013). Transcriptome analysis of *Salicornia europaea* under saline conditions revealed the adaptive primary metabolic pathways as early events to facilitate salt adaptation. PLoS ONE.

[140] Ma J., Zhang M., Xiao X., You J., Wang J., Wang T., Yao Y., Tian C., Li C. (2013). Global transcriptome profiling of *Salicornia europaea* L. shoots under NaCl treatment. PLoS ONE.

[141] Mendis C.L., Padmathilake R.E., Attanayake R.N., Perera D. (2025). Learning from *Salicornia*: Physiological, biochemical, and molecular mechanisms of salinity tolerance. Int. J. Mol. Sci..

[142] Alfheeaid H.A., Raheem D., Ahmed F., Alhodieb F.S., Alsharari Z.D., Alhaji J.H., BinMowyna M.N., Saraiva A., Raposo A. (2022). *Salicornia bigelovii*, *S. brachiata* and *S. herbacea*: Their nutritional characteristics and an evaluation of their potential as salt substitutes. Foods.

[143] Cárdenas Pérez S., Niedojadło K., Świdzinski M., Orzoł A., Strzelecki J., Piernik A., Kačík F., Ďurkovič J. (2025). Cell wall remodeling and polarized light analysis reveal ecotype-specific strategies in *Salicornia europaea* L. with biotechnological applications. Sci. Rep..

[144] Perez S.C., Strzelecki J., Piernik A., Dehnavi A.R., Trzeciak P., Puchałka R., Mierek-Adamska A., Pérez J.C., Kačík F., Račko V. (2024). Salinity-driven changes in *Salicornia* cell wall nanomechanics and lignin composition. Environ. Exp. Bot..

[145] Wang X., Fan P., Song H., Chen X., Li X., Li Y. (2009). Comparative proteomic analysis of differentially expressed proteins in shoots of *Salicornia* europaea under different salinity. J. Proteome Res..

[146] Lv S., Jiang P., Tai F., Wang D., Feng J., Fan P., Bao H., Li Y. (2017). The V-ATPase subunit A is essential for salt tolerance through participating in vacuolar Na^+^ compartmentalization in *Salicornia europaea*. Planta.

[147] Pajuelo E., Romano-Rodríguez E., Mateos-Naranjo E., Flores-Duarte N.J., Rodríguez-Llorente I.D., Redondo-Gómez S. (2025). Halophytes as holobionts: Disentangling the contribution of plant genotype and environmental factors to the associated microbiome of hydro- and xerohalophytes. Curr. Res. Microb. Sci..

[148] Zhang A., Liu Y., Wang F., Li T., Chen Z., Kong D., Bi J., Zhang F., Luo X., Wang J. (2019). Enhanced rice salinity tolerance via CRISPR/Cas9-targeted mutagenesis of the OsRR22 gene. Mol. Breed..

[149] Ly L.K., Ho T.M., Bui T.P., Nguyen L.T., Phan Q., Le N.T., Khuat L.T.M., Le L.H., Chu H.H., Pham N.B. (2024). CRISPR/Cas9 targeted mutations of OsDSG1 gene enhanced salt tolerance in rice. Funct. Integr. Genom..

[150] Wang T., Xun H., Wang W., Ding X., Tian H., Hussain S., Dong Q., Li Y., Cheng Y., Wang C. (2021). Mutation of GmAITR genes by CRISPR/Cas9 genome editing results in enhanced salinity stress tolerance in soybean. Front. Plant Sci..

[151] Yu J., Zhu C., Xuan W., An H., Tian Y., Wang B., Chi W., Chen G., Ge Y., Li J. (2023). Genome-wide association studies identify OsWRKY53 as a key regulator of salt tolerance in rice. Nat. Commun..

[152] Hazzouri K.M., Khraiwesh B., Amiri K.M.A., Pauli D., Blake T., Shahid M., Mullath S.K., Nelson D., Mansour A.L., Salehi-Ashtiani K. (2018). Mapping of HKT1;5 gene in barley using GWAS approach and its implication in salt tolerance mechanism. Front. Plant Sci..

[153] Duan H., Tiika R.J., Tian F., Lu Y., Zhang Q., Hu Y., Cui G., Yang H. (2023). Metabolomics analysis unveils important changes involved in the salt tolerance of *Salicornia europaea*. Front. Plant Sci..

